# Supplementary material for: Exploring the Origins of Association of Poly(acrylic acid) Polyelectrolyte with Lysozyme in Aqueous Environment through Molecular Simulations and Experiments
Source: Polymers (Basel). 2024 Sep 11;16(18):2565. doi: 10.3390/polym16182565 (PMC11434948; doi:10.3390/polym16182565)
Supplement: Supplementary file 1 [file polymers-16-02565-s001.zip › polymers-3154496-supplementary.pdf]

Supporting Information

Exploring the Origins of Association of Poly(acrylic acid) Polyelectrolyte with Lysozyme in Aqueous Environment through Molecular Simulations and Experiments

Maria Arnittali[1,2,3], Sokratis N. Tegopoulos[4], Apostolos Kyritsis[4], Vagelis Harmandaris[1,2,3], Aristeidis Papagiannopoulos[5,*], Anastassia N. Rissanou[5,*]

1. Institute of Applied and Computational Mathematics, Foundation for Research and Technology Hellas, IACM/FORTH, FR-71110 Heraklion, Greece.
2. Department of Mathematics and Applied Mathematics, University of Crete, GR-71409, Heraklion, Crete, Greece.
3. Computation-based Science and Technology Research Center, The Cyprus Institute, Nicosia 2121, Cyprus.
4. National Technical University of Athens, School of Applied Mathematical and Physical Sciences, Athens, Greece.
5. Theoretical & Physical Chemistry Institute, National Hellenic Research Foundation, 48 Vassileos Constantinou Avenue, 11635 Athens, Greece.

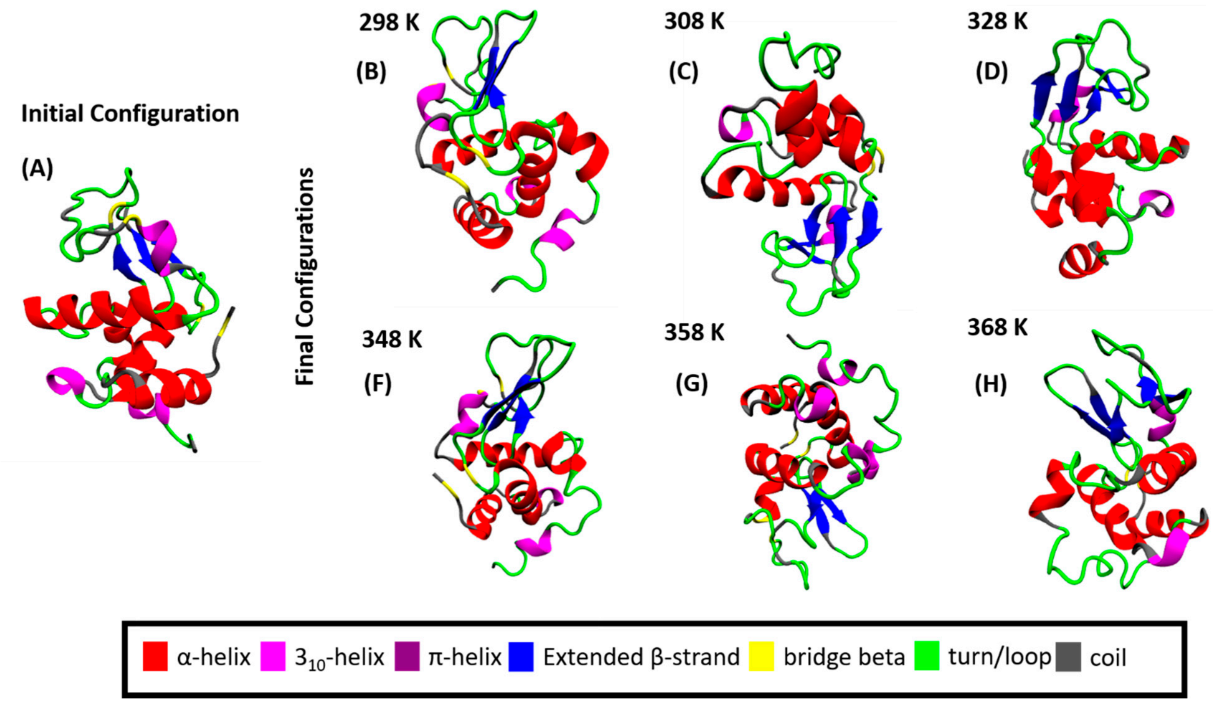

Figure S1. (A) Initial configuration from crystallographic structure of Lysozyme and Final configurations (B),(C),(D),(F),(G),(H) at 298K, 308K, 328K, 348K, 358K, and 368K respectively.

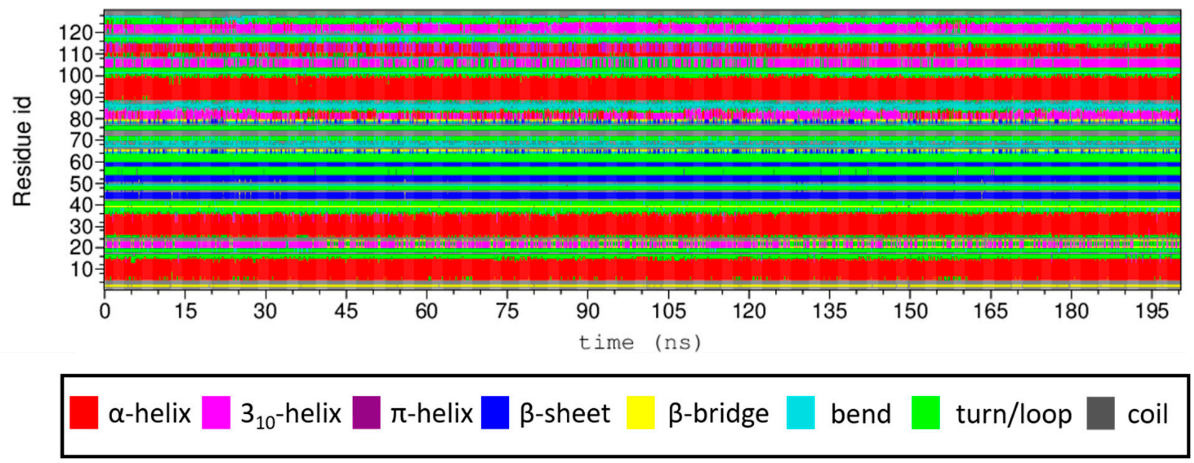

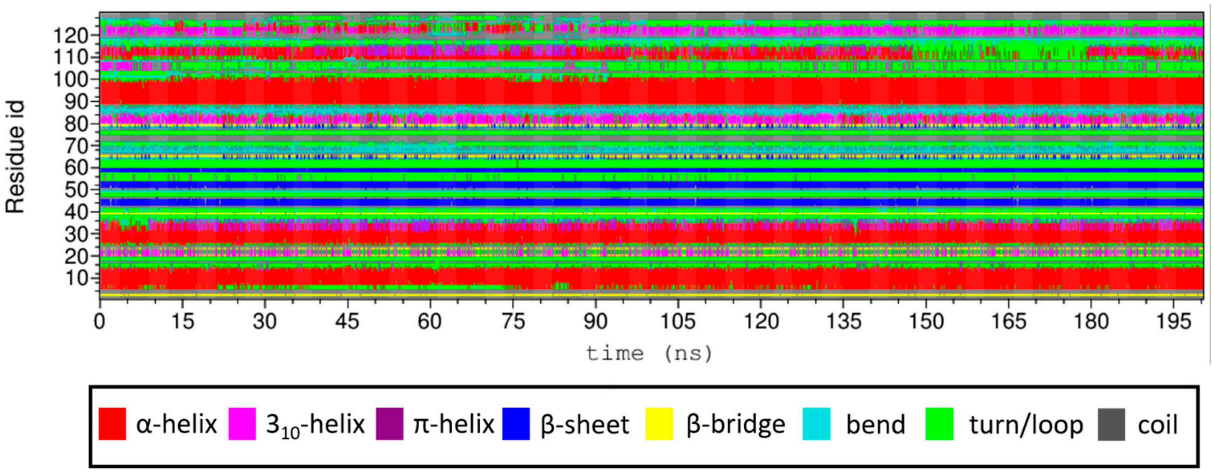

Figure S2: DSSP analysis of Lysozyme at (up) 298K and (down) 368K.

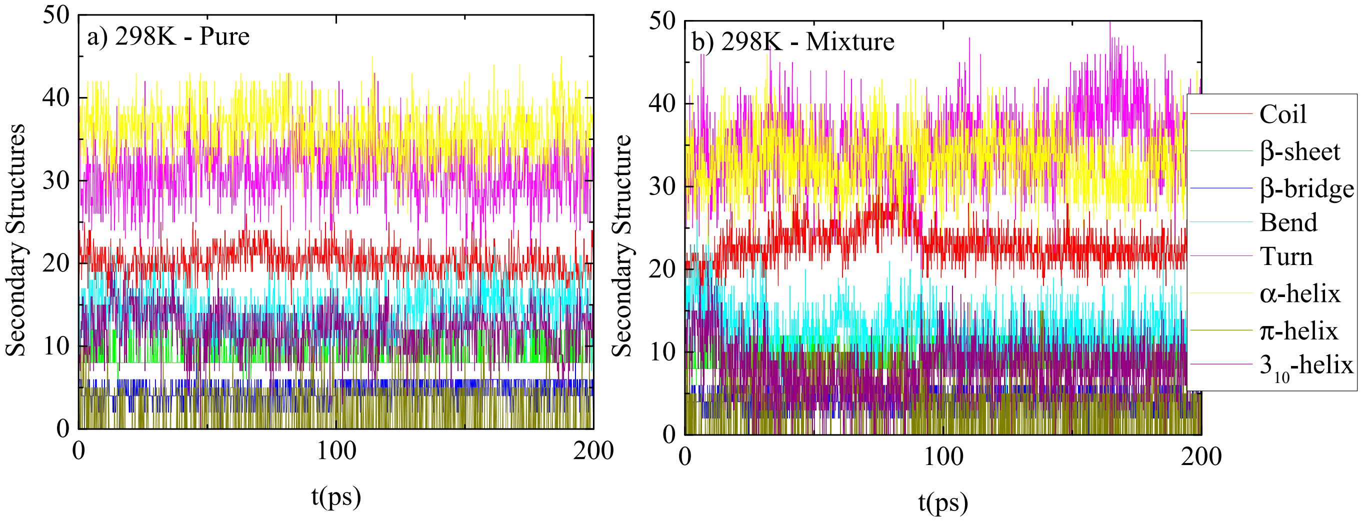

Figure S3: Secondary structure quantification as a function of time for Lysozyme at 298K (a) Pure system and (b) Mixture.

Table S1: Average number of hydrogen bonds over an "equilibrated" part of the trajectory between all-possible pair of molecules in the system at different temperatures.

Systems	HB_{P-P}	HB_{P-W}	HB_{L-L}	HB_{L-W}	HB_{P-L}	T (K)
L_{298}	5.88±2.17	596.58±16.55	103.48±4.02	239.32±8.64	39.29±3.82	298
L_{308}	5.96±2.58	586.82±18.62	95.11±3.99	244.76±8.59	45.39±4.05	308
L_{328}	10.22±2.82	523.87±22.05	100.52±4.69	228.98±8.97	31.73±3.77	328
L_{348}	11.37±3.21	486.31±17.12	94.76±4.70	227.86±10.31	38.74±5.84	348
L_{358}	7.19±2.51	468.05±15.22	98.37±4.64	212.83±9.81	38.89±4.97	358
L_{368}	8.97±2.73	438.06±15.62	98.28±4.72	212.97±9.45	35.11±4.17	368
$L_{Annealing}$	10.28±2.58	508.23±15.12	104.23±4.37	229.83±8.08	39.61±3.36	298

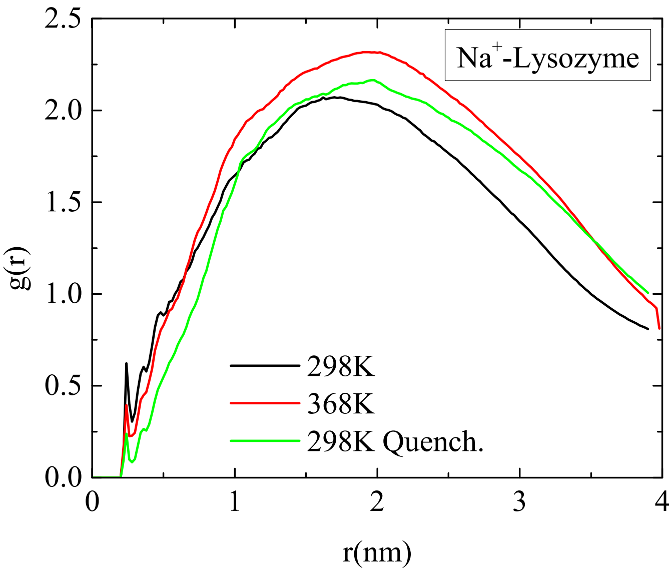

Figure S4: Pair radial distribution functions between Na^+ and protein atoms at 298K, 368K and at 298K after quenching.

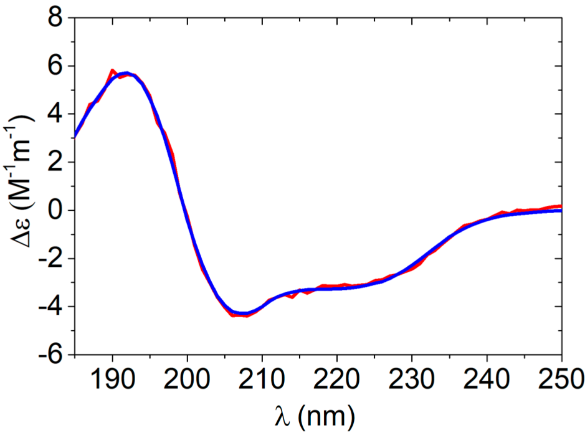

Figure S5: Molar ellipticity of Lys (0.1 mgml-1) at pH 7 (red). Fitting curve is shown in blue.

Table S2. Secondary structure of Lys estimated from ATR-FTIR in % percentage of the separate structural components in Lys and complexes with r_m=0.01 and r_m=0.03 at Lys concentration 1 $mgml^{-1}$.

**Assignment**	***β*-sheet %**	**Random coil %**	***α*-helix**	***β*-turn %**
Wavenumbers (cm^{-1})	1610-1642	1643-1650	1650-1659	1660-1699
Lys pH 7	18±3	-	47±3	35±3
Lys pH 7 thermally treated	24±3	-	22±3	54±3
r_m=0.01 pH 7	27±3	18±3	25±3	30±3
r_m=0.01 pH 7 thermally treated	33±3	18±3	22±3	27±3
r_m=0.03 pH 7	21±3	20±3	26±3	33±3
r_m=0.03 pH 7 thermally treated	40±3	17±3	18±3	25±3

Table S3. Estimation of the secondary structure of lysozyme using FTIR. Results from previous studies.

**Assignment**	***β*-sheet %**	**Random coil %**	***α*-helix**	***β*-turn %**	**References**
Wavenumbers (cm^{-1})	1610-1642	1643-1650	1650-1659	1660-1699	
Lys pH 6.5	21±1	8±0	46±3	26±1	Sadat 2020 [62]

**Assignment**	***β*-sheet %**	**Random coil %**	***α*-helix**	References
Wavenumbers (cm^{-1})	1629±3, 1638±3, 1697±2	1646±2, 1672±2, 1686±1	1656±2	Fu 1999 [61]
Lys pH 7.4	23±3	52±3	27±3	

**Assignment**	***β*-sheet %**	**Random coil %**	***α*-helix**	***β*-turn %**	References
Wavenumbers (cm^{-1})	1623-1637	1646	1655-1660	1668-1691	Krimm 1986 [63]
Lys	20±2	13±2	32±2	31±2	

**Assignment**	***β*-sheet %**	**Random coil %**	***α*-helix**	References
Wavenumbers (cm^{-1})	1615-1637, 1682-1689	1637-1644	1647-1661	Vasita 2012 [64]
Lys	20±5	49±5	31±3	

**Assignment**	***β*-sheet %**	**Random coil %**	***α*-helix**	***β*-turn %**	References
Wavenumbers (cm^{-1})	1624-1642±1, 1691-1696±1,	1648±2	1656-1663±3	1667-16853±2	Kong 2007 [65]
Lys	19	14	40	27	
